# Supplementary material for: Uncovering the causes and socio-demographic constructs of stillbirths and neonatal deaths in an urban slum of Karachi
Source: PLoS One. 2024 Apr 5;19(4):e0298120. doi: 10.1371/journal.pone.0298120 (PMC10997060; doi:10.1371/journal.pone.0298120)
Supplement: S1 File — (PDF) [file pone.0298120.s003.pdf]

Vaccines and other Initiatives to Advance Lives

31-March-2017

Yasir Shafiq
Manager Research
VITAL Pakistan Trust
Karachi

Dear Mr. Yasir Shafiq

001-VPT-IRB-17. Journey through childbirth and newborn survival – an in-depth analysis of perinatal deaths from a low-income community in Karachi, Pakistan as maternal-newborn health intervention coverage was increased

Thank you for submitting your revised application for ethical approval. Your study was reviewed and discussed in IRB meeting. There were no major ethical issues. The study was given an approval for a period of one year with effect from 31-March-2017 For further extension, a request must be submitted along with theannual report.
Any changes in the protocol or extension in the period of study should be notified to the Committee for prior approval. All informed consents should be retained for future reference.

Please ensure that all the national and institutional requirements are met.
Thank you.

Sincerely,

Dr. Fozia Qureshi
Chairperson,
IRB Committee,
Vital Pakistan Trust
