# Supplementary material for: Uncovering the causes and socio-demographic constructs of stillbirths and neonatal deaths in an urban slum of Karachi
Source: PLoS One. 2024 Apr 5;19(4):e0298120. doi: 10.1371/journal.pone.0298120 (PMC10997060; doi:10.1371/journal.pone.0298120)
Supplement: Attachment — Submitted filename: Point-by-point response.docx [file pone.0298120.s004.docx]

PONE-D-23-24729

Uncovering the Causes and Socio-demographic Constructs of Stillbirths and Neonatal Deaths in an Urban Slum of Karachi

Point-by-point response

| **Reviewer comments** | **Author response** |
| --- | --- |
| **Journal requirement** | |
| Thank you for submitting the above manuscript to PLOS ONE. During our internal evaluation of the manuscript, we found significant text overlap between your submission and previous work in the [introduction, conclusion, etc.]. We would like to make you aware that copying extracts from previous publications, especially outside the methods section, word-for-word is unacceptable. In addition, the reproduction of text from published reports has implications for the copyright that may apply to the publications.   Please revise the manuscript to rephrase the duplicated text, cite your sources, and provide details as to how the current manuscript advances on previous work. Please note that further consideration is dependent on the submission of a manuscript that addresses these concerns about the overlap in text with published work.   [If the overlap is with the authors’ own works: Moreover, upon submission, authors must confirm that the manuscript, or any related manuscript, is not currently under consideration or accepted elsewhere. If related work has been submitted to PLOS ONE or elsewhere, authors must include a copy with the submitted article. Reviewers will be asked to comment on the overlap between related submissions (<http://journals.plos.org/plosone/s/submission-guidelines#loc-related-manuscripts)>.]  We will carefully review your manuscript upon resubmission and further consideration of the manuscript is dependent on the text overlap being addressed in full. Please ensure that your revision is thorough as failure to address the concerns to our satisfaction may result in your submission not being considered further. | Thank you for sharing this information and we have taken this very seriously to improve the content we are reporting in this manuscript.  We have now addressed the issue and also passed this new version through Turnitin, the similarity index is less than 5%. |
| In your Data Availability statement, you have not specified where the minimal data set underlying the results described in your manuscript can be found. PLOS defines a study's minimal data set as the underlying data used to reach the conclusions drawn in the manuscript and any additional data required to replicate the reported study findings in their entirety. All PLOS journals require that the minimal data set be made fully available. For more information about our data policy, please see <http://journals.plos.org/plosone/s/data-availability>.  "Upon re-submitting your revised manuscript, please upload your study’s minimal underlying data set as either Supporting Information files or to a stable, public repository and include the relevant URLs, DOIs, or accession numbers within your revised cover letter. For a list of acceptable repositories, please see <http://journals.plos.org/plosone/s/data-availability#loc-recommended-repositories>. Any potentially identifying patient information must be fully anonymized.  Important: If there are ethical or legal restrictions to sharing your data publicly, please explain these restrictions in detail. Please see our guidelines for more information on what we consider unacceptable restrictions to publicly sharing data: <http://journals.plos.org/plosone/s/data-availability#loc-unacceptable-data-access-restrictions>. Note that it is not acceptable for the authors to be the sole named individuals responsible for ensuring data access.  We will update your Data Availability statement to reflect the information you provide in your cover letter. | We have uploaded the minimal data on causes of death |
| **Abstract** | |
| Please add a line on pathways leading to stillbirth and neonatal deaths in the introduction. | Thanks for the feedback, we have added this |
| Please mention the study design for clarification. | We clarified this now, thanks |
| Please add a line about data analysis in the methods | Done, thanks |
| It is unclear whether this was a primary study or a secondary analysis. | This was primary data collection; we added this line |
| It is unclear who reviewed verbal and social autopsies and how scoring was done. | We have added this line as well |
| It is worth adding percentages for health system and community factors. | Agree, we added this |
| Please write the conclusion of your study. | Agree, we added this |
| **Introduction** | |
| Overall, the introduction is good, However, the text can benefit from simplifying sentences, reorganizing information for better flow, and reducing redundancies to enhance readability and clarity. | Thanks for the comments, we restructure it now |
| **Methods** | |
| Please mention the study design (Seems like a mixed method/multimethod study) | Yes, we have described it now on page 4 under study design. |
| Too many dates, I suggest restricting the date to the date of this study | Apologies for that, we have now restricted it to only one. |
| Please mention in a paragraph about the MNCH program in the setting. | Agree, please check page 4 under Integrated MNCH services at Rehri Goth |
| Sample size assumptions are missing. | Please check, we have added it under sample size on page 6 |
| Please specify why 40 narrative interviews for thematic analysis were chosen. | Please check, we have added it under sample size on page 6 |
| Is the VASA tool validated? | Not broadly, we have discussed this as under VASA tool on page 5, para 1 |
| What was the sampling technique? | Thanks, we have added it under sample size and sampling technique on page 6 |
| The data collection method should be elaborated rather than details of training of staff. | Agree, we did it now |
| The first six lines of the “Data analysis” section should be moved to the methodology section. | Agree and done |
| Please describe the interview guides and how the participants identified and approached each other. | Thanks, we have added it under sample size and sampling technique on page 6 |
| It is worth adding details about “Koohi Goth Women's Hospital”, especially in terms of maintaining medical records, and the record retrieval process. | We have described it now under Integrated MNCH services at Rehri Goth on page 5. |
| The section on “Ethical consideration and informed consent procedure” should be simplified in terms of language, and fluency for readability and clarification. | Agree and done |
| **Results** | |
| The first paragraph “Descriptives” needs to be rephrased and avoid long sentences that are not connected for clarification. For example: “A total of 421 events were captured; a complete VASA interview was conducted, and forms were filled for 317 (75.3%) events. Out of 104 with incomplete VASA interview status; interviews refused were 57 (13.5%), caregivers were absent were 18 (4.3%), and shifted were 29 (6.9%).” | Agree and we have simplified it |
| I suggest breaking the results into two main sections: 1) high mortality cluster and 2) low mortality cluster for clarification and readability and easy to follow. | Thanks, we tried to break it under each subheading of the result section for better clarity and readability |
| In the results section present the results only not elaborating or interpreting the findings | Agree and we improved it based on the feedback |
| For privacy and confidentiality, don’t mention the name of the health facility. | Agree, we remove the name/s |
| Quotes used in the results are too long to follow and have little meaning conveyed. | Thanks. We have tried to use the most useful information from the quotes now and also reduced the number |
| **Discussion** | |
| The first 3 lines should be removed because of repetition. | Agree and done |
| Write strengths and limitations in the second paragraph. | Agree and done |
| The conclusion in the discussion should be in sync with the conclusion in the abstract. | Thanks, and done |
| **Figures** | |
| Please add the legend (What color code indicated) | Done |
| There are three figures, but the caption of figure is missing. | We added that |
| What is the purpose of figure three? Should be described in either result of discussed in the discussion section? | Figure three is presenting out thematic analysis and we added more explaining related to this on page 10-11 |
